# Detection and Classification of Bronchiectasis Based on Improved Mask-RCNN

**DOI:** 10.3390/bioengineering9080359

**Published:** 2022-08-01

**Authors:** Ning Yue, Jingwei Zhang, Jing Zhao, Qinyan Zhang, Xinshan Lin, Jijiang Yang

**Affiliations:** 1 Department of Radiology, The Second Hospital, Cheeloo College of Medicine, Shandong University, Jinan 250033, China; yueyue2526@126.com; 2 School of Artificial Intelligence, Beijing University of Posts and Telecommunications, Beijing 100876, China; 18843011036@163.com (J.Z.); zh_qinyan@163.com (Q.Z.); 3 Department of Respiratory and Critical Care Medicine, Shandong Provincial Hospital Affiliated to Shandong First Medical University, Jinan 250021, China; summer6384@hotmail.com; 4 Department of Automation, Tsinghua University, Beijing 100084, China

**Keywords:** bronchiectasis, LDCT, automated scoring, object detection, Mask R-CNN, decision support system

## Abstract

Bronchiectasis is defined as a permanent dilation of the bronchi that can cause pulmonary ventilation dysfunction. CT examination is an important means of diagnosing bronchiectasis. It can also be used in severity scoring. Current studies on bronchiectasis have focused on high-resolution CT (HRCT), ignoring the more common low-dose CT (LDCT). Methodologically, existing studies have not adopted an authoritative standard to classify the severity of bronchiectasis. In effect, the accuracy of detection and classification needs to be improved for practical application. In this paper, the ACER image enhancement method, RDU-Net lung lobe segmentation method and HDC Mask R-CNN model were proposed to detect and classify bronchiectasis. Moreover, a Python-based system was developed: after inputing an LDCT image of a patient’s lung, it can automatically perform a series of processing, then call on the trained deep learning model for detection and classification, and automatically obtain the patient’s bronchiectasis final score according to the Reiff and BRICS scoring criteria. In this paper, the mapping relationship between original lung CT image data and bronchiectasis scoring system was established. The accuracy of the method proposed in this paper was 91.4%; the IOU, sensitivity and specificity were 88.8%, 88.6% and 85.4%, respectively; and the recognition speed of one picture was about 1 s. Compared to a human doctor, the system can process large amounts of data simultaneously, quickly and efficiently, with the same judgment accuracy as a human doctor. Doctors only need to judge the uncertain cases, which significantly reduces the burden of doctors and provides a useful reference for doctors to diagnose the disease.

## 1. Introduction

Bronchiectasis is a common disease caused by the destruction of the bronchial wall or inflammation and disease of the surrounding tissues, resulting in the deformation and expansion of the bronchi. Bronchiectasis can cause severe coughing and sputum, and in severe cases, breathing difficulties and life-threatening symptoms. The main pathogenic factors of bronchiectasis are heredity, bronchial infection, bronchial obstruction and bronchial traction. A 2016 study conducted in Europe by McDonnell et al. showed that the prevalence of bronchiectasis is approximately 5/100,000, and its prevalence and incidence are increasing rapidly [1]. According to the epidemiological survey in China, the prevalence rate of bronchiectasis in Chinese people aged 40 and above is about 1.2% [2]. However, due to misdiagnosis, missed diagnosis and patients’ neglect, the actual prevalence of bronchiectasis is much higher than this figure [3]. Bronchiectasis has a long course, and it is easy to be infected and difficult to recover from it, which seriously affects the quality of life and even endangers the lives of patients, causing serious harm to patients, families and society. Therefore, it is of great significance to study bronchiectasis [4].

At present, the diagnosis of bronchiectasis is mainly based on the observation of lung CT images by doctors. Bronchiectasis in lung CT images is usually characterized by columnar bronchiectasis, thickening of bronchial wall and cystic changes of bronchus. Compared with X-ray chest radio graphs, lung CT images have a higher diagnostic rate for small lesions, especially those smaller than 1 cm. Lung CT scanning is mainly divided into high-resolution CT (HRCT) and low-dose CT (LDCT). Compared with HRCT, the radiation dose of LDCT is lower, which is more suitable for physical examination screening of large-scale population and people who need multiple examinations in a short period of time, and the price is lower, so it is a safe and fast examination method. However, there are few studies on the application of LDCT in bronchiectasis, although the image quality of LDCT can be slightly inferior to the HRCT, but when used in clinical diagnosis of bronchiectasis, these kinds of differences in the quality of imaging can be ignored, and because of the economic, convenient and low radiation, LDCT in the diagnosis of bronchiectasis has great research value.

Object detection is a popular direction of computer vision and digital image processing. It is widely used in robot navigation, industrial detection, medical care and many other fields. It has important practical significance to reduce the consumption of human capital through computer vision. Due to the wide application of deep learning, target detection algorithms have also been developed rapidly [5].

The study in this paper is mainly aimed at the lung low-dose CT data set. Firstly, the data set is collated and annotated, then image enhancement is carried out, and then the pretreatment method of lung lobe segmentation is used. Finally, the deep learning model was trained to detect bronchiectasis and its severity. The whole process was systematic, and the results were visualized. The main contributions of this paper are as follows:

1. The ACER image enhancement method was proposed to improve the detection and classification effect of deep learning model for bronchiectasis.
2. The RDU-Net model was proposed to improve the effect of lung segmentation. At the same time, using RDU-Net for lung lobe segmentation can also improve the deep learning model for bronchiectasis detection and classification.
3. The HDC Mask R-CNN model was proposed to improve the detection and classification of bronchiectasis.

The main research contents of this paper include data collation and annotation, data preprocessing, deep learning model improvement and experimental comparison, scoring method design combined with scoring standards, automatic scoring system design, etc. The system can not only automatically detect and cut the bronchiectasis sites in CT images but also score the severity of bronchiectasis according to the different lung lobes where the bronchiectasis is located according to the scoring method designed in this paper, which effectively helps doctors to judge the disease of bronchiectasis in patients.

The remaining arrangements of this paper are as follows: Section 2 introduces related work. Section 3 introduces the modeling methods. Section 4 presents the results and discussion. Section 5 introduces the system implementation. Section 6 consists of the conclusions and prospects.

## 2. Related Work

### 2.1. Application of Traditional Machine Learning Algorithms in Detection and Classification of Bronchiectasis

Machine learning was first proposed in the 1950s, and it has gone through three major stages. Perceptron and neural network were proposed in the 1980s after it was discovered that neurons are interconnected. Deep neural networks were proposed in the twenty-first century, with the growth of science and technology, the enhancement of computing performance, and the deepening of study, to implement more difficult tasks, but at a larger cost. The models presented in the first two stages are referred to as traditional machine learning.

Artificially designed features of bronchiectasis in medical image data can be extracted and learned with the help of computer vision and machine learning algorithms, allowing for highly accurate medical judgments on the data. As a result, computer vision technology is gaining traction as a modern medical assistive technology. DeBoer et al. developed a universal automatic bronchiectasis scoring system AAI by integrating machine learning methods. They trained a random forest classifier to distinguish between airway and non-airway lungs. AAI is calculated by dividing the sampling area into percentages of airways. The CT images were visually evaluated for bronchiectasis, and the correlation between bronchiectasis and AAI was calculated. When tested and verified, the automated AAI was able to capture bronchiectasis in cystic fibrosis patients and was positively correlated with expert bronchiectasis scores in different scanners and reconstruction protocols. The automatic bronchiectasis score calculated using this method is a sensitive and objective indicator of research and clinical care [6].

Traditional target detection methods need to manually extract feature information to process medical images, so it is inevitable to have incomplete feature information in this process, which leads to a poor recognition effect.

### 2.2. Application of Deep Learning in Detection and Classification of Bronchiectasis

Traditional machine learning methods for classification and detection of medical images require manual feature design and extraction using appropriate methods, limiting machine learning models to learn only around these pre-selected image features, reducing feature learning ability and judgment ability, and limiting the models’ generalization ability. Since AlexNet [7] proposed the method of deep learning in 2012, it has triggered a research upsurge, and it has also been introduced into the field of target detection. Deep learning relies on the model to choose the best features for learning on its own, allowing deep learning models to learn and analyze features that are otherwise overlooked or difficult to extract by humans more effectively. It has also been proven that deep learning models have higher accuracy rates than traditional machine learning models.

With the development of deep learning technology and the wide application of medical imaging and computer technology in the status of medical diagnosis, the use of deep learning technology analysis and processing for medical images gradually became a hot focus of the present study, and in clinical diagnosis and treatment it is a convenient and economic technology, playing an important role. At present, the medical imaging field using deep learning technology mainly includes CT, MRI, X-ray, ultrasound, endoscopy, etc., to study the lesions of lung, brain, stomach, eye and other organs. In the diagnosis of some diseases, the diagnostic system using deep learning technology has reached or even exceeded the diagnostic accuracy of professional human doctors. In the case of the recent novel coronavirus outbreak of pneumonia, a number of deep learning models for the diagnosis of pneumonia assisted by CT images have been proposed and have played an important role. Not only the diagnosis of COVID-19, but other medical imaging methods combined with deep learning techniques, such as the use of deep learning models to detect and classify bronchiectasis in CT images, are also growing and maturing.

In 2016, Guan et al. used unsupervised learning hierarchical clustering analysis to classify bronchiectasis types and identify the clusters that best distinguish the clinical features of bronchiectasis. They used data from 148 patients with stable bronchiectasis to identify four clusters and test the effectiveness of an unsupervised learning model. They also demonstrated that identifying different phenotypes can help researchers better understand the characteristics and prognosis of bronchiectasis [8].

In 2018, Naseri et al. proposed a threshold-based model for extracting airway and vascular features from HRCT images of bronchiectasis patients and calculating the severity score of bronchiectasis patients with cystic fibrosis (CF). The accuracy is higher when compared to other methods [9].

In 2021, Aliboni et al. designed a tool for CT ROI regions detection and classification of bronchiectasis through two convolutional neural networks (Direct-CNN and Serial-CNN). The performance is compared with other architectures proposed in the literature. A total of 19,059 manually selected ROIs were used for training and testing. The accuracy of the second network and the MEAN F1 score were both 0.84. The performance of the first network is slightly lower: the accuracy is 0.81 and the F1 score is 0.82 on average. Finally, it is proved that the established network can accurately detect and classify bronchiectasis [10].

The diagnosis of bronchiectasis mainly uses the object detection algorithm in deep learning. At present, the mainstream object detection algorithms are mainly divided into one-stage and two-stage [11,12]. The main difference between the two is that the two-stage algorithm requires a proposal (a pre-selection box that may contain objects to be inspected) and then carries out fine-grained object detection, while a one-stage algorithm will directly extract features from the network to predict object classification and location. Zhu et al. proposed a fully automated lung CT cancer diagnosis system. They used Faster R-CNN model to detect and grade pulmonary nodules, and their results showed that the performance of their proposed model was comparable to that of experienced physicians at both the nodular level and the patient level [13].

Regardless of traditional machine learning or deep learning methods, previous studies on bronchiectasis mainly focused on the study of HRCT, while the more widely used LDCT was ignored. In addition, due to the sensitivity and particularity of medical image data, deep learning in the medical field, especially the medical image technology for bronchiectasis detection and classification, is rarely studied, and the development rate is very slow. In order to effectively deal with complex medical image data and better realize the combination of deep learning and bronchiectasis detection and classification, more stable, reliable, and universal deep learning methods must be studied.

## 3. Materials and Methods

### 3.1. Data Acquisition, Collation and Annotation

In this paper, we used the data sets based on a study of a medical group’s nationwide large-scale LDCT screening project, from 1 April 2017 to 31 August 2018, choosing seven provinces/cities of 18 years old and above and a total of 22 medical center health check-up crowd LDCT images, and selecting the patients with bronchiectasis data. With multi-slice spiral CT, low-dose scanning was performed. Scanning parameters: tube voltage 80–130 kVP, tube current ≤ 50 mAs, rotation time ≤ 1.0 s, pitch ≤ 1.0, wide field of vision (FOV = L), reconstructed layer thickness 1.0–7.5 mm, reconstructed layer spacing 0.7 mm. The client was supine and held his breath at the end of deep inspiration. The whole lung scan was performed in one breath-hold from the base of the lungs to the apex of the lungs.

The diagnosis and CT score of bronchiectasis were performed back-to-back by two people. In the current CT image severity assessment system of bronchiectasis, the modified Reiff [14] score is widely used in the clinic because of its simplicity and effectiveness and is one of the important items of the BSI (Bronchiectasis Severity Index). The modified Reiff score ≥ 3 was strongly associated with the number of hospitalizations. Meanwhile, the BRICS [15] scoring system launched in 2018 also showed a significant correlation with the decline of FEV1, and the number of acute exacerbations of hospitalization. Although it has not been promoted clinically due to its late launch, it still has a good clinical application prospect. In order to facilitate the score of bronchiectasis, the BRICS score was used in this paper without considering the range of emphysema. The detailed rules of the two kinds of scoring are shown in Table 1 and Table 2 below.

When one or more of the following three criteria were met and tractive bronchiectasis was excluded, the diagnosis of bronchiectasis could be established: 1. The inner diameter of the airway was larger than that of the accompanying pulmonary artery; 2. Distal airway diameter greater than 2 cm from bronchial bifurcation ≥ proximal airway diameter; 3. Bronchi can be seen in lung tissue within 1 cm of costal pleura or mediastinal pleura. If one or more of the radiographers consider that the image does not conform to the diagnosis of bronchiectasis during the image review, they will be reviewed by an associate chief physician specializing in respiratory imaging and make the final diagnosis. The severity of bronchiectasis was divided into three severity grades and rated as 1, 2, and 3. Grade 1 was classified as mild (lumen diameter was 1–2 times of adjacent vessel diameter), 2 was classified as moderate (lumen diameter was 2–3 times of adjacent vessel diameter), and 3 was classified as severe (lumen diameter was more than 3 times of adjacent vessel diameter).

**Figure 1:**
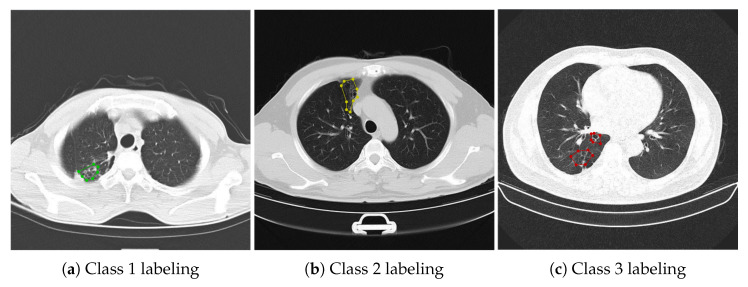
Images of bronchiectasis of different labeled class.

In this study, LDCT images of 120 patients were screened from the above data according to the physical examination results of each center and labeled by doctors according to the above methods and standards. A total of 1992 patients with bronchiectasis were labeled, including 588 patients with category 1, 566 patients with category 2, and 838 patients with category 3. The subdivision for each class is reported in Table 3, and examples of bronchiectasis images with different labeled categories are shown in Figure 1.

### 3.2. Data Preprocessing

Due to the characteristics of the imaging environment and CT scanner itself, the contrast of medical images is generally very low and the tissue structure in the lung is complex. Some images have problems such as unclear structure, blurred edge, and noise. It is not conducive to the clinical diagnosis of bronchiectasis and is not conducive to feature extraction, detection and classification of bronchiectasis by a deep learning model. To solve this problem, this paper uses image enhancement to process lung CT images. Image enhancement can enhance the contrast while making the image details more obvious as much as possible. For this paper, image enhancement is not only beneficial for doctors to judge the location and severity of bronchiectasis based on lung CT images, but also can improve the detection and classification effect of deep learning for bronchiectasis.

Retinex algorithm is a common medical image processing method. The basic principle of Retinex is to divide the image I(x,y) into two components: reflection component R(x,Y) and incident component L(x,Y). R(x,Y) represents the original information of the object, and L(x,Y) represents the brightness image. The illumination component in the image determines the interval size of the maximum and minimum value of the optical signal of an image. The Retinex algorithm first decomposed the image into three channels for processing, and then used a central circumferential function for filtering, which can perform Gaussian filtering on the information of each channel to obtain the light component in the image. The light component is then subtracted from the logarithmic domain to obtain the component that represents the actual reflective properties of the object. Finally, the single-scale Retinex enhanced image was obtained by merging the three-channel data. Its specific expression is as follows:

(1) $$r_{i}\left(x,y\right)=log\left(R_{i}\left(x,y\right)\right)=log\left(\frac{l_{i}\left(x,y\right)}{L_{I}\left(x,y\right)}\right)=log\left(I_{i}\left(x,y\right)\right)-log\left(I_{i}\left(x,y\right)*G\left(x,y\right)\right)$$

where ∗ represents the convolution operation, *i* represents the *i*th color channel, I(x,y) represents the original picture, R(x,y) and r(x,y) represents the reflection component, and G(x,y) is the central circumferential function. The specific expression is:

(2) $$G\left(x,y\right)=\frac{1}{2\pi \delta^{2}}e(-\frac{x^{2}+y^{2}}{2\delta^{2}})$$

**Figure 2:**
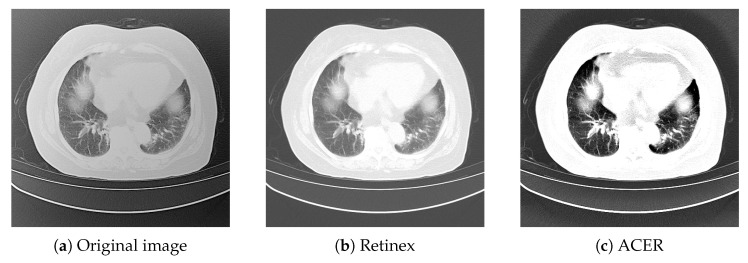
Original image and enhanced image using ACER.

**Figure 3:**
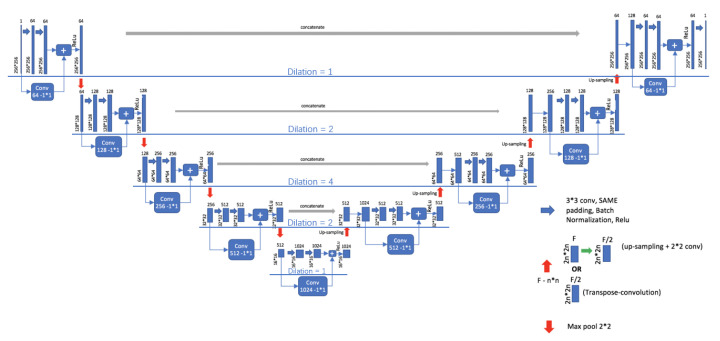
RDU-Net model structure.

**Figure 4:**
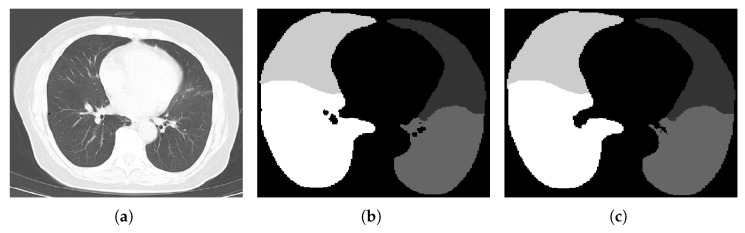
Comparison of two methods of lung lobe segmentation. (**a**) Original image. (**b**) U-Net lung lobe segmentation. (**c**) RDU-Net lung lobe segmentation.

**Figure 5:**
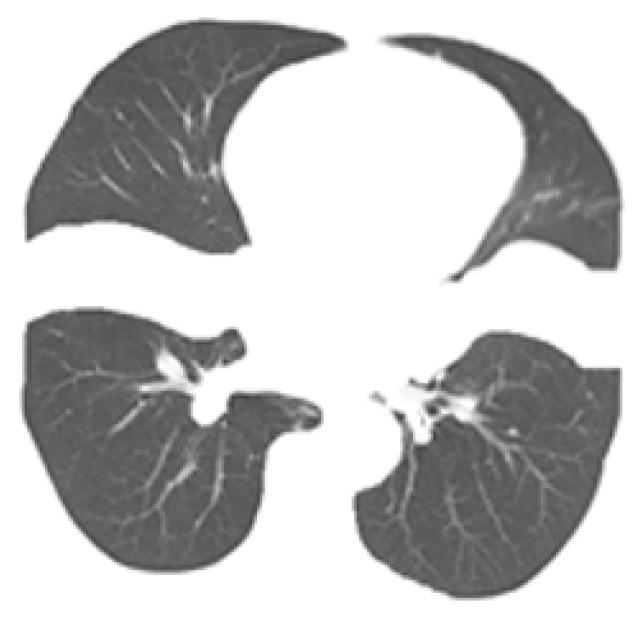
Segmentation image of lung lobe. From left to right from top to bottom as follows: right-up lobe, left-up lobe, right-down lobe, and left-down lobe.

**Figure 6:**
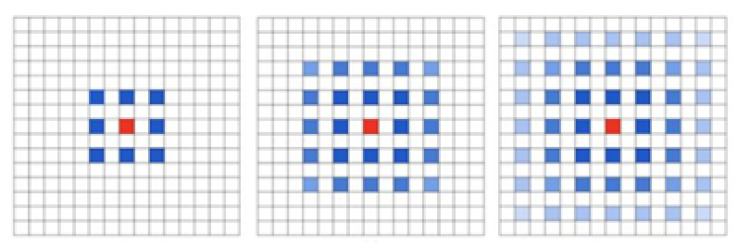
Gridding effect.

**Figure 7:**
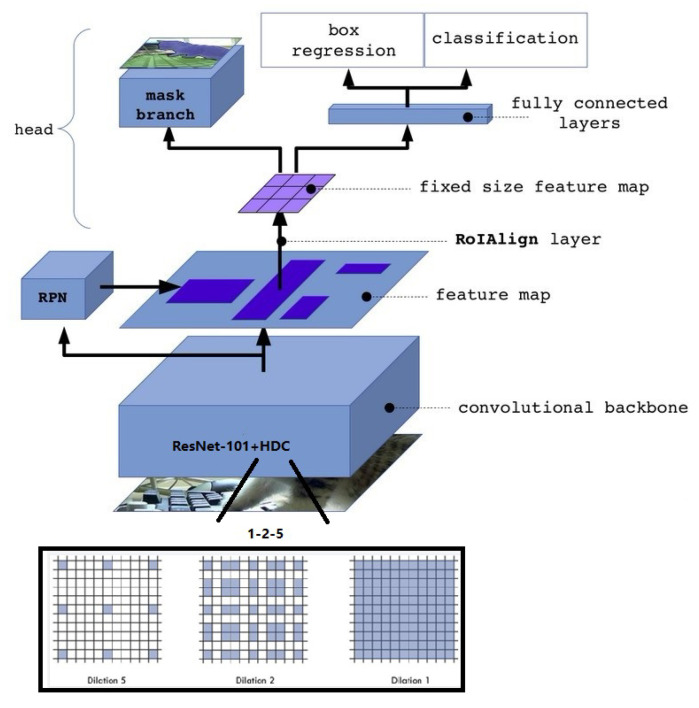
HDC Mask R-CNN structure.

**Figure 8:**
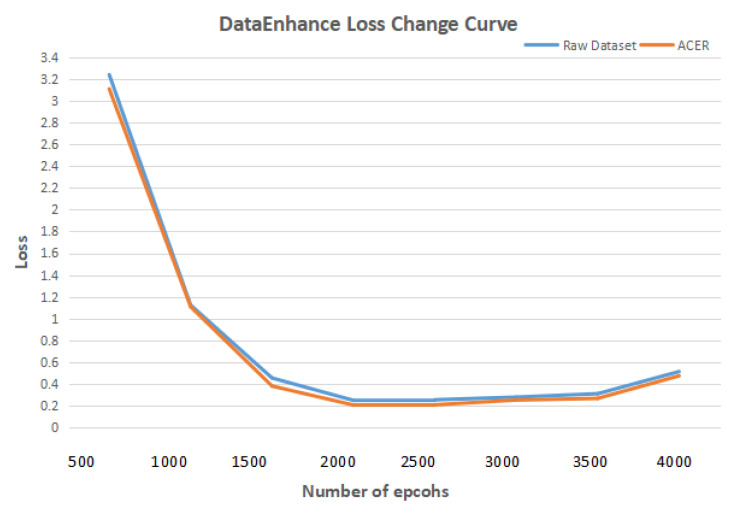
DataEnhance experiment loss change curve.

**Figure 9:**
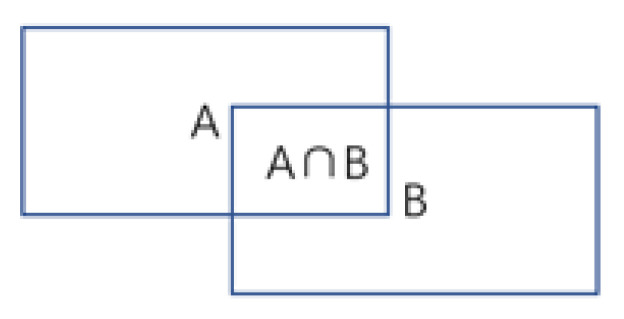
IOU schematic diagram.

**Figure 10:**
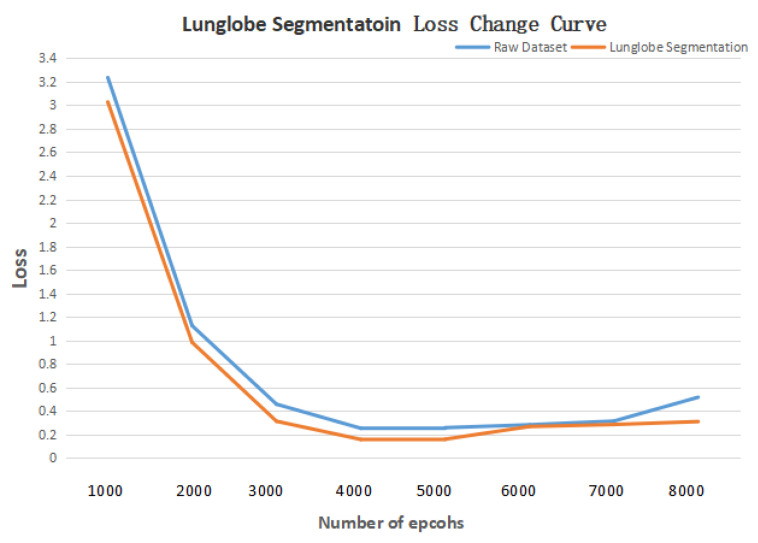
Lung lobe segmentation experiment loss change curve.

Figures 2–10 shows the image enhancement effect after using Retinex algorithm. The Retinex algorithm improves contrast, making the overall image more detailed than the original image, but still not sharp enough. Therefore, this paper proposes an ACER image enhancement method. This method combines the Retinex algorithm with the adaptive contrast enhancement (ACE) algorithm.

The ACER algorithm is based on the principle that the image is first divided into a low-frequency part and a high-frequency part. The low-frequency part is the reverse sharpening mask of lung CT image processed by low-pass filtering, and the high-frequency part is the original image minus the low-frequency part. Then, the contrast gain CG is used as the amplification factor to amplify the high frequency part, and then the amplified high frequency part is added to the low frequency part, namely the anti-sharpening mask. Then, the central circumferential function is used to filter it to obtain the component in the image, and then the component is subtracted in the logarithmic domain to obtain the enhanced image. The lung CT image effect after image enhancement using ACER image enhancement method is shown in Figure 2 below.

### 3.3. Improved U-Net: Residual Module and Dilated Conv U-Net

Lung CT images are not good for deep learning because a lung CT image includes the outer area, the bone, and so on. Therefore, it is necessary to perform lung lobe segmentation for lung CT images. The lung lobe was divided into six regions according to the pulmonary fissure: left upper lobe, left lower lobe, right upper lobe, right middle lobe, right lower lobe, and tongue page, and separated to exclude areas of no interest and serve as a basis for subsequent training and scoring.

In this paper, U-Net is adopted as the basic model of lung segmentation and improved in its basic line. U-Net [16] was proposed by Ronneberger in 2015. Due to its unique structure, U-Net is very suitable for the segmentation task of medical images. Although U-Net is well applied to medical image segmentation, it also has its disadvantages: U-Net uses larger patches and more pooling layers to obtain depth and shallow layer information, which will reduce the accuracy of segmentation, resulting in inaccurate edges and internal holes of segmentationed images. To solve these problems, RDU-Net is proposed in this paper to improve the effect of lung lobe segmentation by applying residual module and dilated convolution to U-Net.The model structure of RDU-Netis shown in Figure 3.

Residual structure is to add a direct connection from input to output into the original convolution structure; this can be done by going directly from input to output at 0 residuals, and the network will not change to original features, so that even if a layer of learning result is bad, performance can still occur under the condition of not falling [17]. When the residual is not 0, feature learning can be carried out normally. Better features are learned from the original input features, while at the same time avoiding over-fitting to increase the nonlinear network [18].

The improvements made in this paper mainly include the following three points.

1. The residual module is combined with the U-Net model. The residual module can make U-Net easier to train and strengthen the information transmission of jump link without degradation, and make edge segmentation more accurate.
2. Dilated convolution is used to replace the original convolution kernel. The principle of dilated convolution is to add holes in normal convolution, and the number of added holes is the dilated rate.
3. When dilated convolution is used, the pooling layer is removed, and then the convolution rate of 3 × 3 convolution in the basic unit structure decreases gradually, and the change in the convolution rate is 1-2-4-2-1.

The structure of the RDU-Net model designed above is shown in Figure 3. This design can avoid the information loss caused by downsampling, expand the receptive field better, and effectively reduce the holes generated in the segmentation process. Table 4 shows the comparison of advantages and disadvantages between U-NET and RDU-NET in segmentation. Figure 4 shows the comparison between the original image and the actual effect of lung segmentation using U-Net and RDU-Net. Figure 5 is the image of each lung lobe after using RDU-Net for lung lobe segmentation.

### 3.4. Improved Mask R-CNN: Hybrid Dilated Conv Mask R-CNN

In this study, a target detection model needs to be trained by using lung CT images that have marked the location and severity of bronchiectasis, so that it can realize automatic detection and severity classification of bronchiectasis in the input images, that is, to judge whether there is bronchiectasis in the input images and mark the location of bronchiectasis. The severity of bronchiectasis in the image was classified according to the designed severity category.

In this paper, Mask R-CNN was used as the basic model to detect and classify bronchiectasis. Mask R-CNN [19] was proposed by He Keming in 2017. Based on Faster R-CNN model [20], Mask R-CNN mainly makes the following three improvements: RoIAlign, full convolutional network FCN and a Mask prediction are added. Although Mask R-CNN has an amazing effect on common data sets such as imagenet, its effect is not ideal for medical image detection tasks, especially for small targets such as bronchus which need to be segmented from lung CT images as in this paper. After the image is input into the model, it should be convolved first and then pooled, which can increase the receptive field but reduce the image size at the same time. Then the upsampling operation is carried out to restore the original size for segmentation and prediction. However, the segmentation targets of medical images, such as bronchiectasis in this paper, are generally very small objects that need to be accurate to pixel level. However, in the process of pooling and upsampling, reducing the size first and then increasing the size will cause the loss of information and affect the final segmentation and detection effect. To solve the above problems, HDC Mask R-CNN (Mask R-CNN with Hybrid dilated convolution structure) is proposed in this paper.

Dilated convolution is adding voids into ordinary convolution. Although the dilated convolution has the advantages of effectively improving the receptivity field, reserving data structures and avoiding downsampling, the discontinuity of the convolution kernel will be caused if the original convolution kernel is simply replaced and multiple dilated convolution rates are superposed. A gridding effect will occur, as shown in Figure 6, that is, some pixels will be missing and not all pixels will be used for calculation. The gridding effect will cause the loss of information, which has a great influence on the pixel-level segmentation task [21]. However, if the dilated convolution with the same large dilated convolution rate is blindly used, it is only effective for the segmentation of large objects, and it does not greatly help to improve the segmentation accuracy of small objects. Therefore, it is crucial to design a suitable structure and distribution of dilated convolution [22].

To solve the above problems, this paper designs a Hybrid Dilated Convolution that meets the following three conditions:

1. Make all the dilates used have mutual quality, so as to avoid the gridding effect.
2. Make the dilated convolution follow the cyclic structure from small to large and then from large to small.
3. Capture multi-scale information by convolution rates of dilates of different sizes at different scales. At the same time, the dilated convolution rate should not be too large, so there are three possible distributions: 1-2-5-2-1, 1-3-5-3-1, and 2-3-5-3-2.

In this paper, the three structures of Mask R-CNN were trained, and the accuracy was compared under the same environment and parameter conditions. Based on the above principles and experimental results, the HDC Mask R-CNN model is proposed in this paper. In the model, the cyclic change structure of dilated convolution rate is 1-2-5-2-1. The reasons why we choose the 1-2-5-2-1 structure will be described later in this paper. Such a structure can pay attention to the features of the whole lung and bronchiectasis at the same time, and obtain larger sensation field and more information without using pooling operation to reduce the image, which can effectively avoid pixel loss and the gridding effect. Table 5 shows the comparison of advantages and disadvantages between Mask R-CNN and HDCMask R-CNN in detection and classification. Its structure is shown in Figure 7.

## 4. Results and Discussion

### 4.1. Experimental Results of Image Enhancement

First, a comparative experiment was designed to verify that using the ACER method to enhance the image enhancement of lung CT images can improve the effect of deep learning in detecting and classifying bronchiectasis. The deep learning model in the comparison experiment uses Mask R-CNN. In the data set, both the initial data set and the data set enhanced by ACER method were used. Other experimental environments and parameters remained unchanged. Figure 8 shows the curve of loss with the increase in the number of epcohs. In terms of experimental evaluation indexes, the classification effect was measured by accuracy rate, and detection effect by IOU. The experimental results are shown in Table 6 and Table 7.

IOU is intersection over union, as shown in Figure 9 below. What IOU does is to calculate the ratio of Intersection and Union of two boundary boxes. The union of two bounding boxes is the entire region occupied by A and B, and the intersection is the overlap region of A and B, so the intersection ratio is the size of the intersection divided by the size of the union. The IOU can be used to measure the accuracy of the detected object. The higher the IOU value, the higher the accuracy.

It can be seen from the table that the ACER image enhancement method proposed in this paper improves the classification accuracy and detection IOU of bronchiectasis by Mask R-CNN by 6 percentage points, that is, it can effectively enhance the contrast and clarity of CT images in view of the shortcomings of CT images such as insufficient contrast and unclear details, and improve the deep learning model to detect and classify bronchiectasis.

### 4.2. Results of Lung Segmentation Experiment

Then a comparative experiment was designed to verify the effectiveness of segmenting method of lung lobe. When training U-Net and RDU-Net, the operating system was Ubuntu18.04 and the environment was cuda9.0, tensorflow1.9, and keras2.2.0. Figure 10 shows the curve of Loss with the increase in training times. It can be seen from the figure that the lowest Loss occurs when the number of epochs is 4000, which is to get the best results. The data set and other parameters used in the training remain the same. U-Net and RDU-Net were used to segment the same CT image test set, respectively, and the IOU of each lobe after segmentation is shown in Table 8 below. It can be seen that the effect of lung segmentation using the RDU-Net model is better than that of U-Net, which can well complete the task of lung segmentation, provide lung images for subsequent bronchiectasis detection and scoring model training, and strengthen the effect of deep learning model detection and classification.

Table 9 and Table 10 compare the classification accuracy and detection IOU of bronchiectasis by deep learning model Mask R-CNN after training on the original data set and the data set after using RDU-Net for lung segmentation. As can be seen from the table, the classification accuracy of bronchiectasis by Mask R-CNN increased by 5 percentage points and detection IOU increased by 10 percentage points after the application of the lung lobe segmentation method, which verified the improvement of the deep learning model classification and detection of bronchiectasis by using RDU-Net lung lobe segmentation.

### 4.3. Detection and Classification of Experimental Results

Table 11 shows the comparison of bronchiectasis classification effects of Mask R-CNN models with three structures trained using ACER and RDU-Net processed data sets. The HDC Mask R-CNN with 1-2-5-2-1 structure has the highest accuracy, which verifies that the HDC Mask R-CNN with 1-2-5-2-1 structure is optimal.

In medical classification tasks, we should not only consider the classification accuracy of detected lesions, but also consider the undetected lesions, namely specificity and sensitivity. To calculate specificity and sensitivity, four concepts need to be defined: True Positive (TP, bronchiectasis detected, and actual bronchiectasis), False Positive (FP, bronchiectasis detected, but no actual bronchiectasis), True Negative (TN, no bronchiectasis detected, and no actual bronchiectasis), and False Negative (FN, no bronchiectasis detected, but actual bronchiectasis). Specificity and sensitivity are defined as:

(3) $$Sensitivity=\frac{TP}{TP+FN}$$

(4) $$Specificity=\frac{TN}{TN+FP}$$

**Figure 11:**
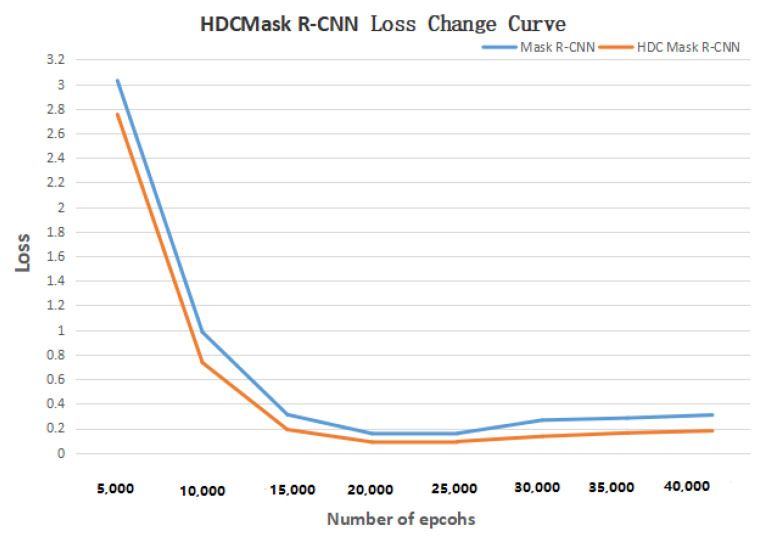
HDC Mask R-CNN loss change curve.

**Figure 12:**
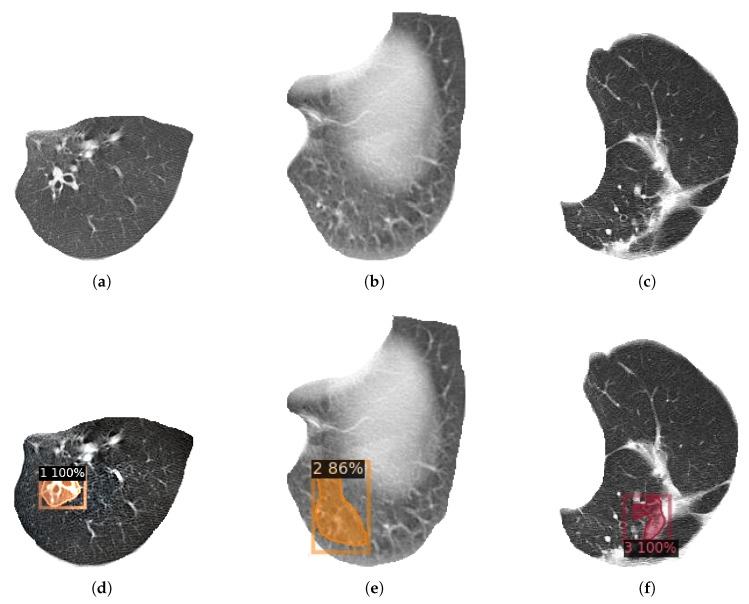
HDC Mas R-CNN detection results for three types of bronchiectasis. (**a**) Class 1 original image. (**b**) Class 2 original image. (**c**) Class 3 original image. (**d**) Class 1 result. (**e**) Class 2 result. (**f**) Class 3 result.

Finally, an experiment was designed to compare the detection and classification effects of HDC Mask R-CNN model and original Mask R-CNN model on bronchiectasis. The experimental data set adopts the data set after image enhancement and lung lobe segmentation. Except for different models, the data set, environment, and other parameters remain the same. Figure 11 shows the curve of Loss with the increase in the number of epochs. Table 12, Table 13, Table 14 and Table 15 are Mask R-CNN and HDC Mask R-CNN using the 1-2-5 2-1 structure, compared in the detection specificity, sensitivity, classification accuracy, and detection IOU of bronchiectasis after training on the data set after using ACER for image enhancement and using RDU-Net for lung lobe segmentation. Figure 12 shows the final detection effect of three types of bronchiectasis.

As can be seen from the table, HDC Mask R-CNN is improved to different degrees than the original Mask R-CNN model in terms of classification accuracy, IOU, specificity and sensitivity. In other words, HDC Mask R-CNN can better detect and classify bronchiectasis in processed lung CT images.

The HDC Mask R-CNN model used in this paper has an accuracy of 91.4% in bronchiectasis classification, with a detection IOU of 88.8%, sensitivity of 88.6%, and specificity of 85.4%. It only takes 1 s to detect one image. Under the premise of high accuracy, it has a fast detection speed and high reference value. Compared with doctors’ manual judgment of bronchiectasis on lung CT images, the speed is greatly improved on the premise of the same accuracy, and a large amount of data can be processed quickly and efficiently at the same time. Because of the high accuracy, the identification results can be used as a very useful reference for doctors to distinguish bronchiectasis. Doctors only need to judge the uncertain samples of the model, which improves the efficiency and reduces the burden of doctors. Figure 12 shows the detection and classification results of three types of bronchiectasis obtained by using the research method used in this paper.

## 5. System Implementation

**Figure 13:**
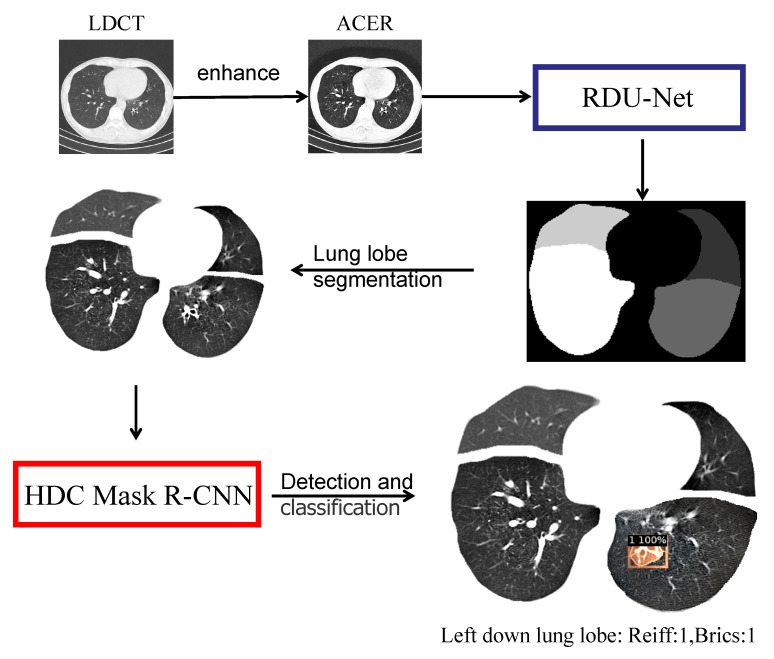
System flow diagram.

The ultimate goal of this study is to implement an automated Python-based system. The patient’s CT image is fed into the system, and the system automatically performs a series of procedures to determine the patient’s bronchiectasis severity score. The specific process of the system is shown in Figure 13 below.

Finally, this paper constructed a Python-based system: Reiff and BRICS automatic scoring system for CT images of bronchiectasis, which can automatically perform a series of operations after inputting LDCT images of patients’ lungs, as follows: The HDC Mask R-CNN model was used for image enhancement and lung segmentation, and the bronchiectasis severity score of the input images was given according to Reiff and BRICS scoring criteria. After a CT image was input, the ACER method was first used for image enhancement, and then RDU-Net was used for lung lobe segmentation. After the segmentation, the images were sent to HDC Mask R-CNN model for detection, and the bronchiectasis position and score of each lung lobe were obtained. Finally, Reiff and BRICS scoring rules were combined. The final bronchiectasis severity score was obtained. As shown in the figure above, the figure is divided into four lobes: upper left, lower left, upper right, and lower right. Among them, no bronchiectasis in the left upper lobe and bronchiectasis in the left lower lobe were classified as 1 with a confidence probability of 100 percent, along with no bronchiectasis in the right upper lobe and no bronchiectasis in the right lower lobe. Therefore, according to Reiff scoring rules and BRICS scoring rules, the final score of the bronchiectasis severity of this image was Reiff 1 point and BRICS 1 point.

The system can not only automatically detect the bronchiectasis site in the CT image after inputting the patient’s CT image, but also can score the severity of bronchiectasis according to certain scoring standards according to the difference of the lung lobe where the bronchiectasis is located, effectively helping doctors to judge the bronchiectasis in patients. Under the condition of ensuring the recognition accuracy, the efficiency is greatly improved, and the classification accuracy is high, which has great reference value.

## 6. Conclusions and Prospects

In this paper, the high-quality data set collected by us was used for the detection and classification of bronchiectasis. Data pretreatment was firstly carried out: in this paper, ACER image enhancement method was first proposed to make the bronchiectasis lesions more obvious. Then, the RDU-Net model of lung segmentation was proposed to provide a basis for the following detection and classification. Then, the HDC Mask R-CNN model was proposed in this paper for the detection and classification of bronchiectasis. Through experimental verification in this paper, the model can well complete the task of detection and classification of bronchiectasis. The accuracy of the model for the classification of bronchiectasis reached 91.4%, while the detection IOU reached 88.8%. The specificity reached 85.4%, and the detection speed was fast. It only took about 1 s to detect a picture, which fully met the needs of medical diagnosis. Finally, we designed and implemented a Python-based Reiff and BRICS automatic scoring system for bronchiectasis in CT images. The system could not only detect the location of bronchiectasis in the input image according to the lung lobe, but also obtain the Reiff and BRICS severity scores of the whole image. This experiment makes full use of modern technology to build a set of automatic classification and scoring systems for bronchiectasis, which has extremely important practical significance for saving the shortage of doctor resources in today’s society and helping people in remote areas get more professional diagnosis. The results of this experiment can be used as a very useful reference for doctors to distinguish branch expansion, which not only greatly improves the efficiency but also reduces the burden of doctors.

Compared with other studies that use deep learning to diagnose bronchiectasis, such as Kannan [23], who used probabilistic neural networks to predict bronchiectasis, this experiment can more specifically detect which lobe of lung bronchiectasis appears in the case of improved detection rate and accuracy. Then, based on this, the overall severity of pulmonary bronchitis can be obtained according to the authoritative scoring system, which makes the evaluation of the severity of bronchitis more rigorous and convincing. Moreover, the system is a set of automatic procedures based on Python, which is easy to operate and convenient for medical staff and patients without relevant professional foundation.

At the same time, this study also has some limitations. First of all, the training data used in the experiment are not enough. If the training data can be increased, the experimental effect will be better. Due to the limited conditions, the hardware equipment used in this experiment is also insufficient, so the training times and model parameters of the experiment are limited.

In the follow-up study, we can further improve the effect of this study from many aspects. One is to label more lung CT images to increase the training set and test set. Increasing the amount of data can significantly improve the training effect of the model. The other is to find better hardware equipment to train the model. Good hardware allows us to design more experiments and significantly improve experimental efficiency. Third, the possibility of optimization can be found in the model structure to better apply to this study, such as adjusting the model structure and optimizing the loss function. Fourth, the process of the experimental system can be further improved and new functions can be added such as perfecting the segmentation part of the lung so that it can separate out the tongue, or designing a new, more comprehensive scoring system.

## Figures and Tables

**Table 1:** Improved REIFF scoring criteria ^1^.

Score	0	1	2	3
Degree of bronchiectasis ^2^	None	Mild ^3^	Moderate ^4^	Severe ^5^

^1^ Six lung lobes (lung 5 lobes + lingual lobes) were scored separately, and the full score was 18 points. ^2^ The degree of bronchiectasis was scored according to the most dilated part of the lung lobes. ^3^ Lumen diameter 1–2 times of adjacent vessel diameter. ^4^ Lumen diameter 2–3 times of adjacent vessel diameter. ^5^ Lumen diameter more than 3 times of adjacent vessel diameter.

**Table 2:** BRICS scoring criteria ^1^.

Score	0	1	2	3
Degree of bronchiectasis	None	Mild ^2^	Moderate ^3^	Severe ^4^
Range of emphysema	None	1–5	>5	

^1^ The total score of bronchiectasis degree and emphysema range was 5 points. ^2^ Lumen diameter 1–2 times of adjacent vessel diameter. ^3^ Lumen diameter 2–3 times of adjacent vessel diameter. ^4^ Lumen diameter more than 3 times of adjacent vessel diameter.

**Table 3:** Original dataset distribution.

	Mild	Moderate	Severe	Total
Quantity	588	566	838	1992

**Table 4:** Comparison of advantages and disadvantages of segmentation models.

Models	Edge Segmentation	Deep Web Degradation Problem	Hole After Segmentation	Receptive Field
U-Net	inaccurate	serious	many	small
Ours	accurate	no	no	bigger

**Table 5:** Comparison of advantages and disadvantages of detection models.

Models	Information Loss	Pixel Using	Gridding Effect	Receptive Field
Mask R-CNN	serious	part	yes	small
Ours	reduce	all	no	bigger

**Table 6:** Comparison of classification accuracy of image enhancement.

Data Enhancement Method	1	2	3	Average Accuracy
Initial Data Set	70.4%	71.2%	75.8%	72.5%
Retinex	72.2%	74.8%	77.6%	74.8%
ACER	77.9%	78.5%	85.3%	80.6%

**Table 7:** Comparison of detection IOU of image enhancement.

Data Enhancement Method	1	2	3	Average IOU
Initial Data Set	61.2%	63.5%	66.3%	63.7%
Retinex	62.4%	65.8%	70.1%	66.1%
ACER	68.4%	70.1%	77.3%	71.9%

**Table 8:** IOU comparative experiment of lung segmentation.

Models	Left Upper	Left Lower	Right Upper	Right Mid	Right Lower	Average IOU
U-Net	91.2%	91.2%	91.6%	91.5%	91.6%	91.4%
RDU-Net	98.2%	98.1%	98.4%	98.3%	98.2%	98.3%

**Table 9:** Lung segmentation model comparative experiment of classification accuracy.

Pretreatment Method	1	2	3	Average Accuracy
Original Image	78.0%	78.5%	85.3%	80.6%
Lung Lobe Segmentation	84.5%	84.6%	86.3%	85.1%

**Table 10:** Lung segmentation model comparative experiment of detection IOU.

Pretreatment Method	1	2	3	Average IOU
Original Image	68.4%	70.1%	77.3%	71.9%
Lung Lobe Segmentation	79.5%	80.2%	84.7%	81.5%

**Table 11:** A comparative experiment on the accuracy of three kinds of HDC structures.

Model Structure	1	2	3	Average Accuracy
1-2-5-2-1	90.9%	91.4%	92.0%	91.4%
1-3-5-3-1	90.5%	91.1%	91.3%	91.0%
2-3-5-3-2	89.9%	90.1%	90.6%	90.2%

**Table 12:** The sensitivity of Mask R-CNN and HDC Mask R-CNN was compared.

Methods	1	2	3	Average Sensitivity
Mask R-CNN	82.2%	82.4%	83.8%	82.8%
HDC Mask R-CNN	88.5%	88.2%	89.1%	88.6%

**Table 13:** The specificity of Mask R-CNN and HDC Mask R-CNN was compared.

Methods	1	2	3	Average Specificity
Mask R-CNN	78.2%	79.5%	82.2%	80.0%
HDC Mask R-CNN	84.6%	85.2%	86.4%	85.4%

**Table 14:** The classification accuracy of Mask R-CNN and HDC Mask R-CNN was compared.

Methods	1	2	3	Average Accuracy
Mask R-CNN	90.7%	91.1%	91.5%	91.1%
HDC Mask R-CNN	90.9%	91.4%	92.0%	91.4%

**Table 15:** The detection IOU of Mask R-CNN and HDC Mask R-CNN was compared.

Methods	1	2	3	Average IOU
Mask R-CNN	87.2%	88.5%	88.9%	88.2%
HDC Mask R-CNN	87.8%	89.2%	89.4%	88.8%

## Data Availability

Not applicable.
